# Genetically Determined Measures of Striatal D2 Signaling Predict Prefrontal Activity during Working Memory Performance

**DOI:** 10.1371/journal.pone.0009348

**Published:** 2010-02-22

**Authors:** Alessandro Bertolino, Paolo Taurisano, Nicola Marco Pisciotta, Giuseppe Blasi, Leonardo Fazio, Raffaella Romano, Barbara Gelao, Luciana Lo Bianco, Madia Lozupone, Annabella Di Giorgio, Grazia Caforio, Fabio Sambataro, Artor Niccoli-Asabella, Audrey Papp, Gianluca Ursini, Lorenzo Sinibaldi, Teresa Popolizio, Wolfgang Sadee, Giuseppe Rubini

**Affiliations:** 1 Psychiatric Neuroscience Group, Department of Neurological and Psychiatric Sciences, University of Bari, Bari, Italy; 2 Department of Neuroradiology, IRCCS “Casa Sollievo della Sofferenza”, San Giovanni Rotondo, Italy; 3 Nuclear Medicine Unit, Department of Internal Medicine and of Public Medicine, University of Bari, Bari, Italy; 4 Clinical Brain Disorders Branch, Genes, Cognition and Psychosis Program, National Institute of Mental Health, National Institutes of Health, Bethesda, Maryland, United States of America; 5 Program in Pharmacogenomics, Department of Pharmacology, College of Medicine, and Division of Biostatistics, College of Public Health, The Ohio State University, Columbus, Ohio, United States of America; 6 IRCCS CSS-Mendel Institute, Rome, Italy; 7 Department of Experimental Medicine, Sapienza University, Rome, Italy; University of Groningen, Netherlands

## Abstract

**Background:**

Variation of the gene coding for D2 receptors (*DRD2*) has been associated with risk for schizophrenia and with working memory deficits. A functional intronic SNP (rs1076560) predicts relative expression of the two D2 receptors isoforms, D2S (mainly pre-synaptic) and D2L (mainly post-synaptic). However, the effect of functional genetic variation of *DRD2* on striatal dopamine D2 signaling and on its correlation with prefrontal activity during working memory in humans is not known.

**Methods:**

Thirty-seven healthy subjects were genotyped for rs1076560 (G>T) and underwent SPECT with [^123^I]IBZM (which binds primarily to post-synaptic D2 receptors) and with [^123^I]FP-CIT (which binds to pre-synaptic dopamine transporters, whose activity and density is also regulated by pre-synaptic D2 receptors), as well as BOLD fMRI during N-Back working memory.

**Results:**

Subjects carrying the T allele (previously associated with reduced D2S expression) had striatal reductions of [^123^I]IBZM and of [^123^I]FP-CIT binding. *DRD2* genotype also differentially predicted the correlation between striatal dopamine D2 signaling (as identified with factor analysis of the two radiotracers) and activity of the prefrontal cortex during working memory as measured with BOLD fMRI, which was positive in GG subjects and negative in GT.

**Conclusions:**

Our results demonstrate that this functional SNP within *DRD2* predicts striatal binding of the two radiotracers to dopamine transporters and D2 receptors as well as the correlation between striatal D2 signaling with prefrontal cortex activity during performance of a working memory task. These data are consistent with the possibility that the balance of excitatory/inhibitory modulation of striatal neurons may also affect striatal outputs in relationship with prefrontal activity during working memory performance within the cortico-striatal-thalamic-cortical pathway.

## Introduction

Susceptibility to schizophrenia is explained for the largest fraction by genetic variation [1]. While the specific genes conferring risk for schizophrenia are still undetermined, several studies and meta-analyses point to the potential involvement of the gene for dopamine D2 receptors (*DRD2*) [2], [3]. Moreover, several lines of evidence suggest involvement of the dopamine system and of D2 signaling in the pathophysiology of schizophrenia [4]–[6]. Indeed, all antipsychotics available on the market block dopamine D2 receptors (even though other receptors may also be involved). Phenomenologically, schizophrenia is characterized by cognitive deficits, in particular in the working memory domain [4]. Working memory deficits in schizophrenia have been associated with dysfunction of the prefrontal cortex [7], [8] and of the dopamine system [4], [6]. Indeed, several authors have hypothesized that altered working memory performance and related prefrontal activity can be part of a systems level pathophysiological mechanism also involving dopamine D2 receptors in the striatum [4], [6]. The key anatomical and molecular mechanisms regulating the relationship between *DRD2* genetic risk, dopamine dysregulation of D2 signaling, and working memory dysfunction remain undetermined.

A large series of studies in animals indicates that prefrontal neuronal activity during performance of working memory tasks is regulated by dopamine [9]. In the prefrontal cortex both D1 and D2 receptors are involved in working memory, with the latter more specifically implicated in the response phase [9]. In this regard, genetically modified mice lacking D2 receptors exhibit behavioral working memory deficits and reduced activity of the prefrontal cortex during treatment with D1 agonists [10]. Moreover, behavioral performance and prefrontal neuronal activity during working memory (WM) performance are also regulated by dopamine and D2 receptors in the striatum, a key node within the cortico-striatal-thalamic-cortical circuit [11]. In this regard, an experiment inducing developmental over-expression of striatal D2 receptors demonstrated specific working memory deficits and altered prefrontal activity to D1 stimulation [12]. These experiments in animals indicate that genetically modified striatal D2 signaling has systems-level effects in dopamine modulation of prefrontal cortical activity and related behaviors. Data in humans have been consistent with these studies demonstrating that infusion of D2 receptor agonists or antagonists is respectively associated with relative improvement or deterioration of working memory performance [13] as well as of prefrontal and striatal activity [14], [15]. Other imaging studies in humans have also demonstrated that striatal dopamine and D2 signaling are correlated with prefrontal activity or behavioral performance during different cognitive operations [16]–[18].

D2 receptors in the striatum are both pre- and post-synaptic and affect the final output of the striatum to the thalamus, providing an important regulation of prefrontal cortex stimulation [19]. At present, however, it is unknown whether genetic determination of pre- and post-synaptic striatal D2 signaling as modulated by dopamine is a mechanism contributing to neuronal activity in prefrontal cortex in humans. The D2 receptor gene (*DRD2*) codes for two isoforms, D2S (short) and D2L (long). D2L receptors mainly mediate post-synaptic signaling. D2S receptors mainly serve as auto-receptors on pre-synaptic neurons [20], even though they are also found on post-synaptic neurons [21]. Moreover, pre-synaptic D2 receptors strongly contribute to physically regulate density and activity of the dopamine transporter (DAT) [22]–[29]. An intronic *DRD2* polymorphism (*rs1076560*, G>T) is associated with mRNA splicing [30]. More specifically, the minor T allele is associated with relatively reduced expression of D2S in prefrontal cortex and striatum as well as with altered activity of the striato-thalamic-prefrontal pathway during WM in healthy subjects [30] and in schizophrenia [31]. However, the effect of this SNP on prefrontal cortical activity may be also because of indirect effects via the striatum [11]. The effects of this genetic variant on pre- and post-synaptic signaling of dopamine in the striatum are not known. In this study in healthy humans, we hypothesized that this functional *DRD2* variant, rs1076560, would be associated: with differential binding of [^123^I]IBZM measured with SPECT, which reflects availability of post-synaptic D2 receptors [32]; with differential binding of [^123^I]FP-CIT measured with SPECT, which reflects availability of pre-synaptic DAT [33]; and with how a factor score of the two radiotracers identifying striatal D2 signaling would predict prefrontal activity measured with BOLD fMRI during performance of a working memory task. Moreover, in an effort to test the relative specificity of these associations, subjects were also genotyped for *COMT* Val^158^Met genotype (rs4680). COMT is a key enzyme for dopamine catabolism in prefrontal cortex but not in striatum [34], [35] and this polymorphism alters activity of the enzyme in association with prefrontal function [36].

## Materials and Methods

### Participants to [^123^I]FP-CIT SPECT, [^123^I]IBZM SPECT, and BOLD fMRI Studies

Thirty-seven healthy subjects (16 males, mean age±SD 23.5±3.0 years) participated to [^123^I]FP-CIT SPECT. Thirty-two of these subjects underwent [^123^I]IBZM SPECT and twenty-eight also underwent Blood Oxygen Level Dependent (BOLD) functional Magnetic Resonance Imaging (fMRI) during performance of the N-Back task. Exclusion criteria included history of significant drug or alcohol abuse (no active drug use in the past year), head trauma with loss of consciousness, and any significant medical condition. Parental socio-economical status (Hollingshead Scale 40.4±14.9), handedness (Edinburgh Inventory 0.74±0.4), and total IQ (WAIS-R, 108.1±14.8) were measured. The present study was approved by the local IRB. After complete description of the study to the subjects, written informed consent was obtained.

#### Genotype determination

*DRD2* rs1076560 genotypes were determined as in [29]–[31]. SNP rs1076560 was analyzed with allele-specific PCR primers as described [37] or SNaPshot (Applied Biosciences (ABI), Foster City CA) [38]. Consistent with the distribution observed in earlier studies [38], no *DRD2 TT* subjects were observed in this sample. *COMT* Val^158^Met genotype (rs4680) was determined as a restriction fragment length polymorphism after PCR amplification and digestion with *Nla*III [39], [40]. The allelic distribution of *DRD2* and *COMT* was in Hardy Weinberg equilibrium (*DRD2* df 1, chi^2^ = 2.1, p>0.1, *COMT* df 2, chi^2^ = 1.3, p>0.4).

#### Acquisition of SPECT data

Each subject was injected intravenously with an average of 150 MBq (range 111–186 MBq) of commercially available [^123^I] FP CIT or [^123^I]IBZM radiotracer (GE Healthcare, Amersham, UK) [41]–[43]. These two radiotracers bind to dopamine transporters [33] or D2 receptors [32], respectively. Potassium Iodide solution (Lugol) was administered at least 3 hours before and 12 hours after radiopharmaceutical injection to block thyroid uptake of free radioactive iodide. Images were acquired 3–6 h after [^123^I]FP-CIT injection [44] or 1.5 hours after [^123^I]IBZM injection [45]. A dual-head gamma camera (Infinia, General Electric) equipped with parallel-hole, low-energy high-resolution collimators was used. SPECT data were acquired using the following parameters: 128×128 matrix, rotation of 360°, 60 view, 6° view angle, 45 s for projection. Slice thickness was 3.68 mm, acquisition time was 22 minutes; total brain counts >1 million were achieved in all examinations. Reconstruction was performed by filtered back-projection with a Butterworth filter (cut-off frequency: 0.3 cycle/cm, 10th order) to provide transaxial slices that were attenuation corrected. Attenuation correction was performed according to Chang's method, (attenuation coefficient: 0.12 cm^−1^), after manually drawing an ellipse around the head contour [46]. System spatial resolution (full width at half-maximum) at a radius of rotation of 15.9 cm is 11 mm, as reported elsewhere [47]. For analysis of striatal radiotracer uptake, slices were reoriented parallel to the canthomeatal line.

#### Processing of SPECT data

The irreversible binding characteristics and the stability of regional [^123^I]FP-CIT and of [^123^I]IBZM uptake have been shown to allow estimation of the specific-to-nondisplaceable equilibrium partition coefficient (V3″), which is proportional to free transporter or receptor density (Bmax) [48], [49]. V3″ can be calculated as indicated earlier [48], [50]. Under equilibrium conditions between a compartment with specific binding and a compartment representing nonspecifically bound and free activity, V3″ is proportional to Bmax given that the dissociation constant and the volume of distribution of the nonspecifically bound and free activity compartment (V2) are relatively invariant. The occipital region was selected as the background region because 1) the density of dopamine D2 and DAT proteins is negligible compared with the striatum [51], [52]; 2) this region can be identified with greater reliability than the cerebellum [53]; 3) in humans [^123^I] IBZM activity in the occipital region is equal to the nonspecific activity in the striatum [54]. Therefore, as in earlier studies [53], [55], [56], the occipital region was used to model non-specifically bound and free activity compartment. V3″ can be calculated in all voxels with the formula as in [50]:
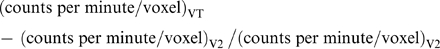
where VT represents specific binding, and V2 the nonspecifically bound and the free activity compartment.

Image transformation, calculation of V3″ and statistical analysis was performed using SPM5 (Wellcome Department of Cognitive Neurology, London, UK). V2 was calculated with a ROI of the occipital lobe from the WFU Pickatlas (http://fmri.wfubmc.edu/cms/software#PickAtlas) [57]–[59]. Since parametric images of [^123^I]FP-CIT and [^123^I]IBZM V3″ lack anatomical detail, an indirect approach was employed for spatial normalization as detailed in [50], [60]. Briefly, the raw [^123^I]FP-CIT and [^123^I]IBZM SPECT data of each subject were normalized on the SPECT template in MNI (Montreal Neurological Institute) space [50] with a 12-parameter affine transformation of the raw data onto the template image followed by estimation of the nonlinear deformations between the applied images. A mean image of the previously normalized raw data acquisitions was then computed and used as a template image. For each individual SPECT acquisition, a parametric V3″ image was calculated. The raw data image was transformed to the template image and the resulting transformation parameters were then applied to the corresponding subject's parametric V3″ image. The spatially normalized parametric images were convolved with a gaussian kernel (6×6×6 mm) for smoothing.

#### Statistical analyses of SPECT data

Two sample T tests were used within SPM5 to evaluate potential differences between genetic groups with a statistical threshold p<0.005, with further correction for multiple comparisons within ROIs in putamen obtained with the WFU_PickAtlas tool, p = 0.05.

Binding of [^123^I] FP-CIT and of [^123^I] IBZM is associated with protein density and affinity for the radiotracer as well as with the relative concentration of endogenous dopamine occupying these proteins [61], [62]. Moreover, there is an extensive literature detailing the interaction between dopamine transporters and D2 receptors [22]–[29], [63]. Therefore, we hypothesized that the effects of *DRD2* genotype on striatal dopamine D2 signaling would be additive and therefore identifiable with a measure reflecting the shared and not the unique variance derived by the two radiotracer binding measures. This index derived by factor analysis would reflect individual dopamine D2 signaling and could be used for correlation with fMRI activity. Therefore, we used a factor analysis approach using a principal component analysis (PCA, total variance used) as a reduction method [64] to combine binding data from the two radiotracers into a single factor. We performed a factor analysis using Principal component Analysis (PCA) with STATISTICA (StatSoft, Tulsa, Oklahoma). [^123^I]FP-CIT and [^123^I]IBZM V3″ data were extracted in each subject from a basal ganglia ROI, which included bilateral caudate and putamen. ROIs were identified using the WFU PickAtlas software (Functional MRI Laboratory at the Wake Forest University School of Medicine, http://www.rad.wfubmc.edu/fmri) [57]–[59]. One principal component (PC) that is a linear combination of the two binding measures was estimated. This PC represents average dopamine D2 signaling weighted by the variance of the binding measures. For each subject we calculated a factor loading, which indicates the weight of each subject bindings in the PC. PC loadings were then used as predictors in random effects analyses to identify potential relationships with the fMRI data in SPM (see below).

ω^2^ is an effect size measure which estimates the proportion of variance in a dependent measure accounted for by independent categorical variables in the population from which the sample was drawn. Thus, we used ω^2^ to measure the amount of variance accounted for by *DRD2* genotype. ω^2^ is given by the equation: 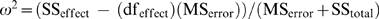.

### BOLD fMRI Data Acquisition and Processing

#### N-Back working memory paradigm

During fMRI, all subjects completed a blocked paradigm of the N-Back task with a 2-Back working memory condition and a non-memory guided control condition 0-Back [40], [65]. This paradigm has been extensively used to evaluate activity of prefrontal cortex. “N-back” refers to how far back in the sequence of stimuli the subject had to recall. The stimuli consisted of numbers (1–4) shown in random sequence and displayed at the points of a diamond-shaped box. There was a visually paced motor task which also served as a non-memory guided control condition (0-Back) that simply required subjects to identify the stimulus currently seen. In the working memory condition, the task required recollection of a stimulus seen two stimuli (2-Back) previously while continuing to encode additionally incoming stimuli. Performance data were recorded as the number of correct responses (accuracy) and as reaction time.

#### BOLD fMRI acquisition parameters

Each subject was scanned with a 3T MR scanner (GE) with a gradient-echo echo-planar imaging (EPI) sequence using the following parameters: 20 contiguous axial slices, slice thickness = 5 mm, echo time = 30 msec, repetition time = 2000 msec; field of view 24 cm; matrix 64×64, voxel size after normalization = 3.75 mm isotropic [40], [66]. We used a simple block design in which each block consisted of eight alternating 0-Back and 2-Back conditions (each lasting 30 seconds), obtained in 4 min and 8 sec, 120 whole-brain fMRI volumes. The first four scans of the time series were acquired to allow the signal to reach a steady state and were not included in the final analysis.

#### BOLD-fMRI image analysis. Preprocessing and statistical analyses

Analysis of the fMRI data was completed using statistical parametric mapping (SPM5; http://www.fil.ion.ucl.ac.uk/spm). Images for each subject were realigned to the first volume in the time series to correct for head motion (<2.5 mm of translation, <1.5° rotation), spatially normalized into a standard stereotactic space (Montreal Neurological Institute, MNI, template) using a 12 parameter affine model and smoothed to minimize noise and residual differences in gyral anatomy with a Gaussian filter, set at 10 mm full-width at half-maximum. Voxel-wise signal intensities were ratio normalized to the whole-brain global mean. Each experimental condition was modeled with a box car convolved with the hemodynamic response function (HRF) at each voxel. Predetermined condition effects at each voxel were calculated using a t-statistic, producing a statistical image for the contrasts of 2-Back versus 0-Back (N-Back). All these individual contrast images were then used in second-level random effects models at the group level. ANOVA was used to evaluate the main effects of working memory and of *DRD2* genotype. Then, we performed separate linear regression analyses for the two groups of subjects (GG and GT) within SPM5. First, we entered the single subject contrasts (2Back>0Back) with [^123^I] IBZM or [^123^I] FP-CIT binding (from the anatomical ROIs differentiating the two genotype groups) as predictors. These analyses do not exclude that the biological effects of *DRD2* genotype can result by additive effects of genetic variants on both pre- and post- synaptic compartments of D2 systems that would be captured by a D2 signaling factor (as detailed above). Thus, we also regressed separately for the two genotype groups the single subject contrasts (2Back>0Back) with factor scores from the factor analysis of the SPECT data of each individual subject as predictors. All statistical maps were thresholded at a level of *p*<0.005 uncorrected, with further FWE small volume correction for multiple comparisons with α = 0.05 using a 10 mm radius sphere centered around coordinates in prefrontal cortex published in previous studies of working memory. These coordinates included: x −20, y 8, z 56 [65]; x −32, y 14, z 38 [67]; x −52, y 26, z 20 [68]; x 38, y 34, z 11 [69]. The studies used to perform small volume correction of our data were selected based on the characteristics of the working memory task that we have used [65], [67], [69], as well as on the aim of the present study (i.e. to investigate the impact of variability in dopamine genes on modulation of the functional activity of the prefronto-striatal network, [68]). Because we did not have *a priori* hypotheses regarding the activity of brain regions outside of the prefrontal cortex, we used a statistical threshold of p = 0.05, corrected for multiple comparisons across all voxels, for these whole-brain comparisons. For anatomical localization, statistical maxima of activation were converted to conform to the standard space of Talairach and Tournoux.

#### Polynomial regression to identify the correlation between behavior, BOLD fMRI, and SPECT data

Because previous evidence supports the hypothesis that the relationship between working memory behavioral performance with dopamine signaling and prefrontal activity is non-linear [9], [70], we performed polynomial regression to assess this relationship in our data. A second order (quadratic) polynomial model was fitted to the data separately in the two genotype groups: behavioral performance at 2-Back was the dependent variable; the dopamine D2 signaling factor score (see above) and BOLD fMRI activity in prefrontal cortex (from clusters correlated with the factor score itself) were the independent variables. The F-statistic was used to compare the goodness of fit of quadratic relative to linear model by comparing the ratio of partitioned variances in the two genotype groups.

## Results

### Demographics and Working Memory Performance Results

The two genotype groups did not differ for any of the demographic measures (See also Supplemental Table S1, all p>0.1) or for working memory behavioral performance (% correct responses GG = 83.4±14.3, GT = 88.1±15.4; Reaction Time GG = 512.4±219.1 msec, GT = 562.0±289.5; all p>0.2) thus allowing us to examine the association of genotypes with brain activity and dopamine signaling independent of behavioral variation in this sample.

### SPECT Results

**Figure 1:**
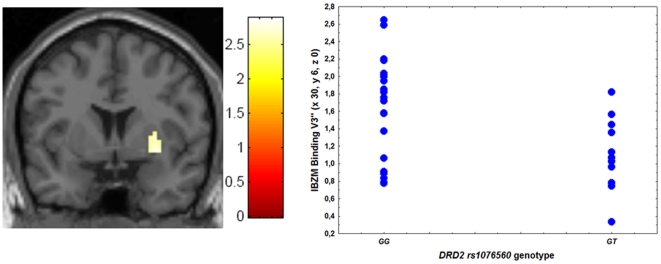
Association between *DRD2 rs1076560* genotype and [^123^I]IBZM binding. Coronal section of the effect of *DRD2 rs1076560* genotype (GG>GT) on [^123^I]IBZM specific binding (V3″) in right putamen (left) and relative scatterplot of individual data points from the cluster differentiating the two groups (right).

**Figure 2:**
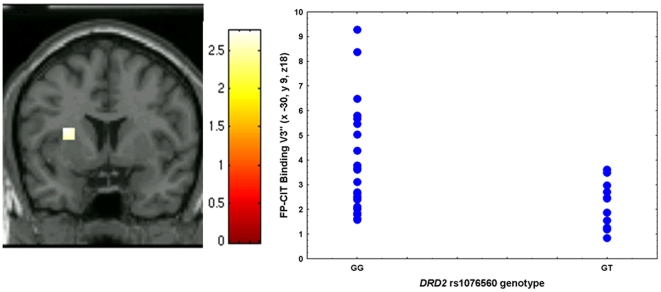
Association between *DRD2 rs1076560* genotype and [^123^I]FP-CIT binding. Coronal section of the effect of *DRD2 rs1076560* genotype (GG>GT) on [^123^I]FP-CIT specific binding (V3″) in left putamen (left) and relative scatterplot of individual data points from the cluster differentiating the two groups (right).

Two-sample t test in SPM5 demonstrated that subjects homozygous for the G allele had significantly greater specific binding of [^123^I]IBZM in right putamen (GG N = 21, GT N = 11, Z = 2.68; k = 7; p = 0.05, corrected; x 30, y 6, z 0, Figure 1) compared with GT subjects. *DRD2* genotype explained 20% of the variance in [^123^I]IBZM binding as indicated by ω^2^. Similarly, GG subjects also had significantly greater specific binding of [^123^I]FP-CIT in left putamen (GG N = 26, GT N = 11, Z = 3.1; k = 13; p = 0.03, corrected; x −30, y 9, z 18, Figure 2). *DRD2* genotype explained 10% of [^123^I] FP-CIT binding variance.

Factor Analysis demonstrated that both data from [^123^I]IBZM and [^123^I]FP-CIT basal ganglia ROIs load into one factor (GG N = 21, GT N = 11, Factor loading for [^123^I] FP-CIT = 0.76, factor loading for [^123^I] IBZM = 0.76, Eigenvalue 1.16, 58% Total Variance) which presumably reflects dopamine D2 signaling.

In an effort to test the specificity of the association with *DRD2*, SPECT data were also grouped and analyzed for *COMT* Val^158^Met genotype (rs4680). Because of the small number of Met homozygous subjects, Met/Met and Met/Val subjects were grouped together. Two sample t tests in SPM5 demonstrated no significant association of *COMT* genotype with both [^123^I]IBZM (Met carriers N = 19; Val/Val N = 8) and [^123^I]FP-CIT data (Met carriers N = 20; Val/Val N = 10), even after lowering the statistical threshold to p<0.01, uncorrected (data not shown).

### fMRI Results

#### Effect of the 2-Back working memory task

As expected from previous studies with the N-Back task [7], [29], [31], [39], [40], [65], [71], [72], ANOVA within SPM5 demonstrated that performance of the 2-Back working memory condition was associated with activity in a distributed network of brain regions including the prefrontal cortex, the parietal cortex, and the striatum bilaterally. Regions that also survived correction for multiple comparisons include the left middle frontal gyrus (x −29, y 13, z 54, BA 6/8 and 9, Z = 6.79, k = 159, corrected p = 0.000), the left inferior frontal gyrus (x −51, y 15, z 19, BA 44/45, Z = 5.55, k = 83, p = 0.000) and the right middle frontal gyrus (x 40, y 40, z 15, BA 10/46, Z = 5.42, k = 66, p = 0.000).

#### Effect of *DRD2* genotype

Consistent with earlier studies [29]–[31], ANOVA within SPM5 indicated that, despite similar behavioral performance, GT subjects tend to have greater activity in prefrontal cortex (x 33, y −1, z 45, middle frontal gyrus, BA 6, Z = 3.13, k = 41, p = 0.001; x 63, y 8, z 7, inferior frontal gyrus, BA 44, Z = 3.36, k = 16, p = 0.0001) and anterior cingulate (x 10, y 16, z 40, anterior cingulate, BA 32, Z = 3.27, k = 21, p = 0.001) compared with GG subjects. However, none of these results survived correction for multiple comparisons.

#### Relationship between fMRI and SPECT data

**Figure 3:**
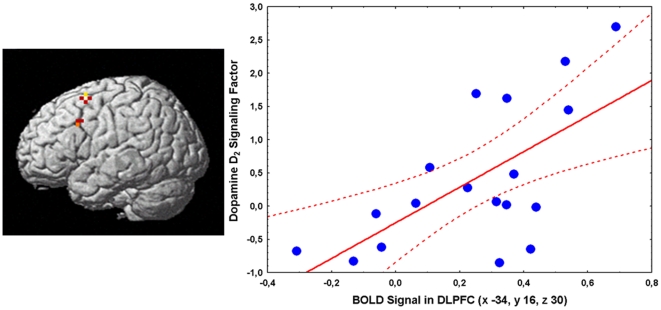
Correlation between BOLD fMRI activity in prefrontal cortex and the striatal dopamine D2 signaling factor in GG subjects. 3D rendering of the correlation between the striatal dopamine D2 signaling factor and BOLD fMRI activity during the 2-Back WM task in GG subjects (left) with the relative scatterplot of the correlation in prefrontal cortex showing individual data points (right).

**Figure 4:**
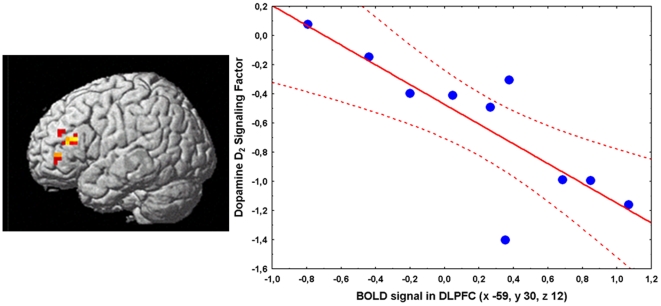
Correlation between BOLD fMRI activity in prefrontal cortex and the striatal dopamine D2 signaling factor in GT subjects. 3D rendering of the correlation between the striatal dopamine D2 signaling factor and BOLD fMRI activity during the 2-Back WM task in GT subjects (left) with the relative scatterplot of the correlation in prefrontal cortex showing individual data points (right).

Regression analyses in SPM5 demonstrated a direct correlation between [^123^I] IBZM binding in right putamen with prefrontal cortex in GG subjects (x −38, y 10, z 37, k = 3, Z = 3.1, p = 0.02, corrected) and a negative correlation in GT subjects (x −60, y 26, z 16, k = 12, Z = 3.1, p = 0.05, corrected). However, no correlation was found in either group between [I^123^] FP CIT binding and BOLD activity during performance of the N-Back working memory task. Also, **r**egression analyses in SPM5 demonstrated a positive relationship between the factor score obtained from SPECT data and BOLD activity in prefrontal cortex in GG subjects (N = 18; x −26, y 10, z 54, BA 6, Z = 3.6, k = 5, p = 0.007 corrected; BA 9 x −34, y 16, z 30; Z = 2.98, k = 8, p = 0.03, corrected, Figure 3)) and a negative one in GT subjects (N = 10; BA 46, Z = 3.36, k = 12, p = 0.03, corrected, x −59, y 30, z 12; BA 46 Z = 2.77, k = 5, p = 0.09, corrected, x 37, y 34, z 16, Figure 4; see also Supplemental Table S2 for further results).

In GG subjects, there was a strong statistical trend for a quadratic relationship between 2-Back percent correct responses with the striatal dopamine D2 signaling factor score and prefrontal activity during working memory as measured with fMRI (R^2^ = 0.45, F = 2.67, df = 4,13, p = 0.07; F-statistic for quadratic vs. linear model (4,13) = 6.44, p = 0.004; Univariate Results: factor score^2^ t = 2.5, p = 0.02; BOLD activity in BA9 x −34, y 16, z 30^2^ t = 1.9, p = 0.07, Figures S1 and S2). In GT subjects, no such relationship was evident (R^2^ = 0.22, F = 0.3, df = 4,5, p = 0.8).

## Discussion

The results of the present study with multimodal imaging in humans demonstrate that *DRD2* rs1076560 genotype predicts striatal [^123^I]IBZM and [^123^I]FP-CIT binding and the direction of the correlation between a factor score reflecting striatal D2 signaling with prefrontal activity during performance of working memory. More specifically, carriers of the T allele of rs1076560, known to be associated with relatively reduced D2S, had reduced [^123^I]IBZM and [^123^I]FP-CIT binding. Consistent with its anatomical distribution, with a relatively specific effect of the *DRD2* genotype and with an earlier study which was published while the present paper was being reviewed [73], *COMT* rs4680 genotype did not demonstrate any association with SPECT data. Importantly, the present data also indicate that *DRD2* genotype is associated with qualitatively different relationships between [^123^I]IBZM binding and the striatal factor score **with** prefrontal activity during working memory. GG subjects have a positive correlation which is also weakly predictive of behavioral performance, whereas in GT subjects the correlation is negative. Earlier studies from our group had provided *in vitro*, *post mortem* and *in vivo* evidence for the functionality of this SNP and for its association with activity of the working memory network [30], [31]. In the present study we provide evidence for a systems level genetically determined mechanism correlating striatal D2 signaling with alteration of prefrontal activity during working memory.

Even though the specificity for the two D2 isoforms is not known, [^123^I]IBZM is believed to bind to post-synaptic D2 receptors [17], [32]. [^123^I]IBZM binding can be modulated by endogenous dopamine and by receptor density, affinity, and internalization [53], [74], [75]. The *rs1076560* G allele has been associated with greater D2S expression, but not with total density of D2 receptors [30]. Thus, reduced [^123^I]IBZM binding in GT subjects can reflect reduced D2S receptors, which are also found post-synaptically [21]. Our [^123^I]-IBZM results are also consistent with earlier studies investigating the association between *DRD2* polymorphisms and D2 binding with different radiotracers [76]–[78]. These polymorphisms are in high linkage disequilibrium with rs1076560 and these earlier studies have reported an average 30% reduction in D2 binding based on genotype. Our data indicate that GT subjects have an average 34% reduction in [^123^I]-IBZM binding compared with GG subjects. Resting state [^123^I]FP-CIT measures pre-synaptic binding to DATs [33]. Given the robust literature providing indication of the physical and functional relationship between DATs and pre-synaptic D2 receptors [28], [29], reduced [^123^I]FP-CIT binding in GT subjects may be because of reduced pre-synaptic D2S. An alternative interpretation of both SPECT datasets is also possible. Because GT subjects have reduced pre-synaptic D2S which inhibits dopamine release [19], reduced [^123^I]IBZM and [^123^I]FP-CIT binding may be because of greater steady-state levels of dopamine competing with the radiotracer. Consistent with these contentions and with knowledge that the main factor contributing to a relationship between pre-synaptic DATs and post-synaptic D2 receptors is dopamine itself, factor analysis of both SPECT datasets identified a factor that may reflect dopamine D2 signaling (the major element in common likely affecting binding of the two radiotracers).

*DRD2 rs1076560* genotype was also differentially associated with the correlation between putative striatal dopamine D2 signaling (as identified with the factor score) and prefrontal activity during working memory, which was positive in GG and negative in GT subjects. A plausible cellular mechanism for these effects is the differential contribution of dopamine D2S and D2L receptors in modulation of glutamate and GABA transmission on striatal output. Both D2 isoforms participate in pre-synaptic inhibition of striatal GABA transmission, while D2S is preferentially involved in modulation of glutamate release [21]. Therefore, modulation of excitatory and inhibitory transmission in the striatum is tightly linked to balance of the two isoforms [21]. In fact, GABA spiny neurons, which account for the large majority of neuronal populations in striatum, only fire action potentials when depolarized by glutamate released by afferents from the cortex [79]. Since *DRD2 rs1076560* genotype alters the relative expression of the two isoforms, our data are consistent with the possibility that the balance of excitatory/inhibitory modulation of striatal neurons may also affect striatal outputs and the relationship with prefrontal activity during working memory.

Since the present study is in healthy subjects, no direct conclusion can be drawn about schizophrenia. However, some speculations as to the relevance of the present findings to schizophrenia can be discussed. A long debated issue in the pathophysiology of schizophrenia is whether subcortical dopamine dysregulation is a primary phenomenon or rather it is associated with prefrontal cortical dysfunction [4], [80]–[82]. Indeed, heritability of both these phenotypes has been demonstrated [83]–[87]. Several studies and meta-analyses point to potential involvement of *DRD2* in susceptibility to schizophrenia. Genome-wide linkage meta-analyses have pointed to involvement of 11q [88]. Metanalyses of case-control studies indicate SNPs rs1801028 (Cys^311^Ser), rs2283265, and rs6277 (C^957^T) [2], [3] as implicated in risk for schizophrenia. Other recent family-based and case control studies have also implicated these SNPs [89], [90]. All these SNPs are linked with rs1076560 in various degrees of LD. Given these earlier studies in the literature and the present findings, it is possible to speculate that risk for schizophrenia may involve the effects of rs1076560 on striatal dopamine signaling and its relationship with prefrontal activity. This speculation is also consistent with the above cited animal studies indicating that developmentally regulated over-expression of striatal D2 receptors is associated with behavioral working memory deficits and with altered activity of the prefrontal cortex [12].

A limitation of the present study needs to be addressed. The time interval between the two SPECT scans was two weeks. Although it would have been preferable that the SPECT and fMRI scans were performed closely in time, this was not always the case and the time interval between the two SPECT scans and the fMRI scan was variable. On average, 25.6±3.6 months elapsed between these scans. Earlier studies have demonstrated good reproducibility for [^123^I] IBZM (as good as 6.5±5.2 [91], [92] and for [^123^I] FP CIT (5.53±4.12%/0.89, [93]). As for fMRI, reproducibility has been investigated in several studies using different working memory paradigms. Studies have reported good reproducibility of Dorsolateral Prefrontal cortex activation in healthy subjects (Intra Class Correlation (ICC = 0.81) coefficient [94]). Similar results have also been reported by other investigators [95], [96]. These earlier studies suggest that it is less likely that the time interval between scans has significantly influenced the results. Moreover, it is important to underline that the relationships identified in this study reflect known neurobiological pathways. Also, there was no statistically significant difference between the two genotype groups in time elapsed between SPECT scans and fMRI (t = 0.09, p = 0.9) which may have unduly influenced the differential correlations. Finally, SPECT and PET studies of DAT and of D2 receptors have demonstrated between 3 and 7% decrease of radiotracer binding per decade, especially after 40 years of age [97]–[101]. Since our healthy subjects are definitely younger than 40 (mean age±SD 23.5±3.0), we believe physiological dopamine signaling decline did not significantly affect our analyses over the time interval of the study. Thus, we interpret these findings as being consistent with stability of fMRI and SPECT measures and as suggesting that they reflect neurobiological mechanisms.

Another point that needs some discussion is that, at the chosen statistical threshold corrected for multiple comparisons, we failed to find a main effect of rs1076560 genotype on prefrontal activity during performance of working memory in the present dataset. In three earlier studies, we had already demonstrated association of rs1076560 with prefrontal activity during performance of the N-Back working memory task in healthy subjects with sample sizes as large as N = 142 [29]–[31]. In the present study, the sample included 28 healthy subjects. Despite the smaller sample size, GT subjects had greater activity in prefrontal cortex and anterior cingulate before correction for multiple comparisons. Earlier studies have demonstrated that larger sample sizes are generally needed for imaging genetics studies using corrected statistical thresholds [102]. Therefore, the present results suggest that the lack of a statistically significant main effect of rs1076560 is likely associated with the relatively small sample size of the present fMRI study. Moreover, the main objective of the present fMRI data was to evaluate statistically significant *DRD2* modulation of the relationship between striatal dopamine D2 signaling with prefrontal activity during performance of working memory which should not be affected by the corrected significance of the above mentioned *DRD2* effect.

Our data are consistent with earlier studies about genetic effects on modulation of the relationship between mesencephalic dopamine and prefrontal cortex activity [103]. Indeed, the ventral tegmental area in the mesencephalon may be implicated in these mechanisms. Our data also provide evidence for a genetic signature that affects striatal dopamine D2 receptor signaling and its relationship with WM prefrontal activity, a mechanism potentially involved in schizophrenia and other different brain disorders [4].

## Supporting Information

Table S1 Demographics of all subjects included in the study divided by rs1076560 genotype.
(0.03 MB DOC)

Table S2 Results from the correlation between 2-Back WM activity and the basal ganglia SPECT factor score that were not corrected for multiple comparisons. No clusters survived the statistical threshold in the inverse correlations.
(0.04 MB DOC)

Figure S1 Relationship between behavioral performance and dopamine D2 signaling. Scatterplot of the non-linear relationship in GG subjects between working memory behavioral performance and the factor score extracted from both SPECT data sets in striatum.
(0.51 MB TIF)

Figure S2 Relationship between behavioral performance and prefrontal activity. Scatterplot of the non-linear relationship in GG subjects between working memory behavioral performance and prefrontal activity during working memory as measured with BOLD fMRI.
(0.50 MB TIF)
