# Supplementary material for: Genetically Determined Measures of Striatal D2 Signaling Predict Prefrontal Activity during Working Memory Performance
Source: PLoS One. 2010 Feb 22;5(2):e9348. doi: 10.1371/journal.pone.0009348 (PMC2825256; doi:10.1371/journal.pone.0009348)
Supplement: Table S1 — Demographics of all subjects included in the study divided by rs1076560 genotype. (0.03 MB DOC) [file pone.0009348.s001.doc]

**Supplemental Table S1.**

|  | **GG** | **GT** |
| --- | --- | --- |
| **n (sex)** | **26 (11M)** | **11 (5M)** |
| **Age** mean (sd, years) | 24.0 (3.0) | 22.5 (3.1) |
| **Hollingshead** | 42.8 (16.2) | 36.3 (10.0) |
| **Handedness** | 0.6 (0.4) | 0.7 (0.4) |
| **IQ** | 109.35 (14.0) | 105.11 (17.3) |
