# Supplementary material for: Genetically Determined Measures of Striatal D2 Signaling Predict Prefrontal Activity during Working Memory Performance
Source: PLoS One. 2010 Feb 22;5(2):e9348. doi: 10.1371/journal.pone.0009348 (PMC2825256; doi:10.1371/journal.pone.0009348)
Supplement: Table S2 — Results from the correlation between 2-Back WM activity and the basal ganglia SPECT factor score that were not corrected for multiple comparisons. No clusters survived the statistical threshold in the inverse correlations. (0.04 MB DOC) [file pone.0009348.s002.doc]

**Supplemental Table S2.**

| **Region (Brodmann’s area)** | **Talairach coordinates (*x,y,z*)** | **k** | ***Z*value** | ***p(uncorrected)*** |
| --- | --- | --- | --- | --- |
| *DRD2* GG: positive correlation |  |  |  |  |
| Right Cingulate Gyrus BA24 | 4 -2 35 | 10 | 3.04 | 0.001 |
| Left Middle Frontal Gyrus BA9 | -41 31 30 | 3 | 2.65 | 0.004 |
| *DRD2* GT: negative correlation |  |  |  |  |
| Right Middle Frontal Gyrus BA11 | 45 36 -14 | 19 | 2.95 | 0.002 |
| Right Inferior Parietal Lobule BA7 | 45 -68 45 | 4 | 2.82 | 0.002 |
| Right Middle Frontal Gyrus BA10 | 8 66 4 | 4 | 2.80 | 0.003 |
| Left Middle Frontal Gyrus BA10 | -38 43 -6 | 7 | 2.65 | 0.004 |
