# Supplementary figures and images for: Genetically Determined Measures of Striatal D2 Signaling Predict Prefrontal Activity during Working Memory Performance
Source: PLoS One. 2010 Feb 22;5(2):e9348. doi: 10.1371/journal.pone.0009348 (PMC2825256; doi:10.1371/journal.pone.0009348)

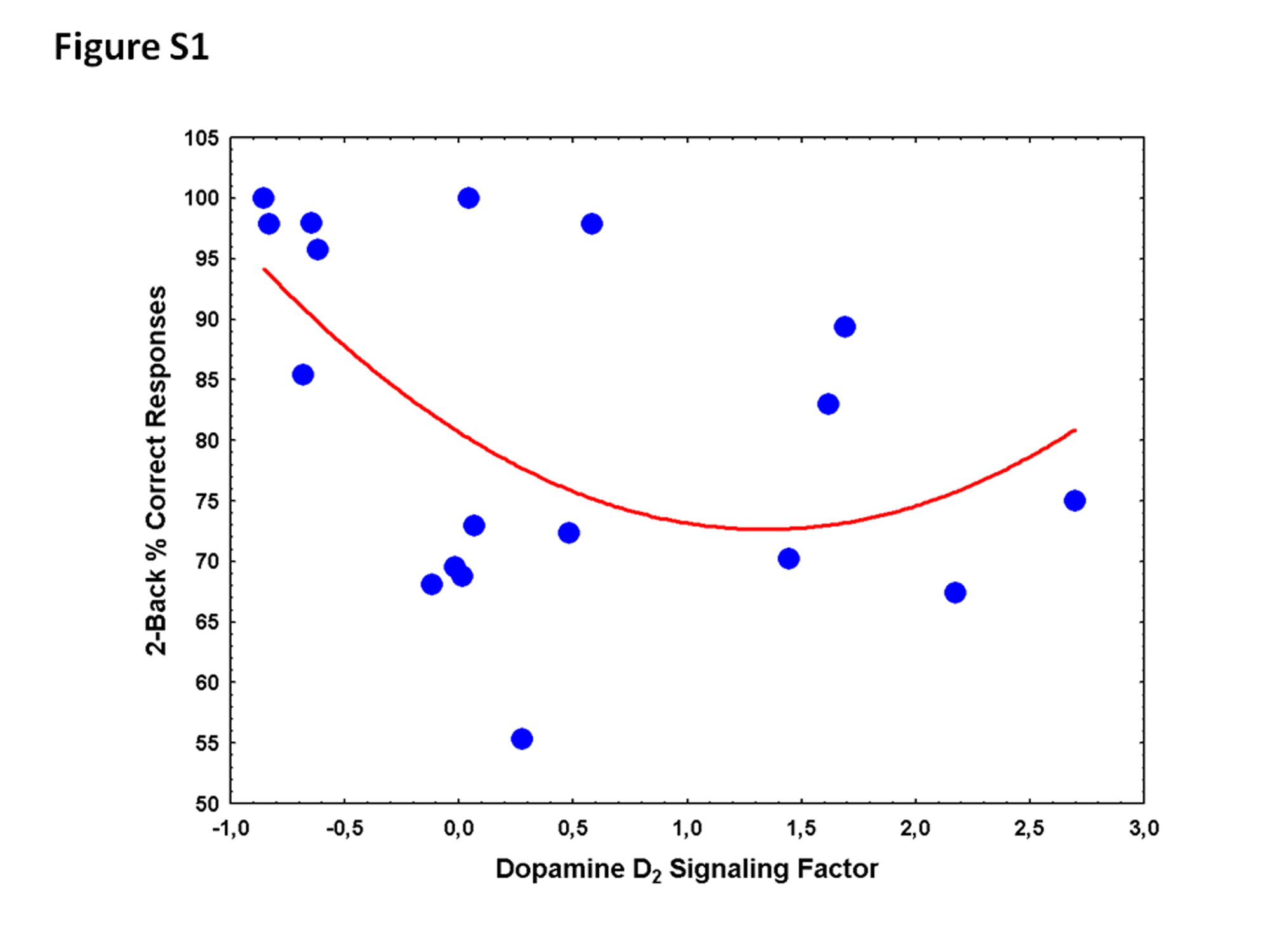

Supplement: Figure S1 — Relationship between behavioral performance and dopamine D2 signaling. Scatterplot of the non-linear relationship in GG subjects between working memory behavioral performance and the factor score extracted from both SPECT data sets in striatum. (0.51 MB TIF) [file pone.0009348.s003.tif]

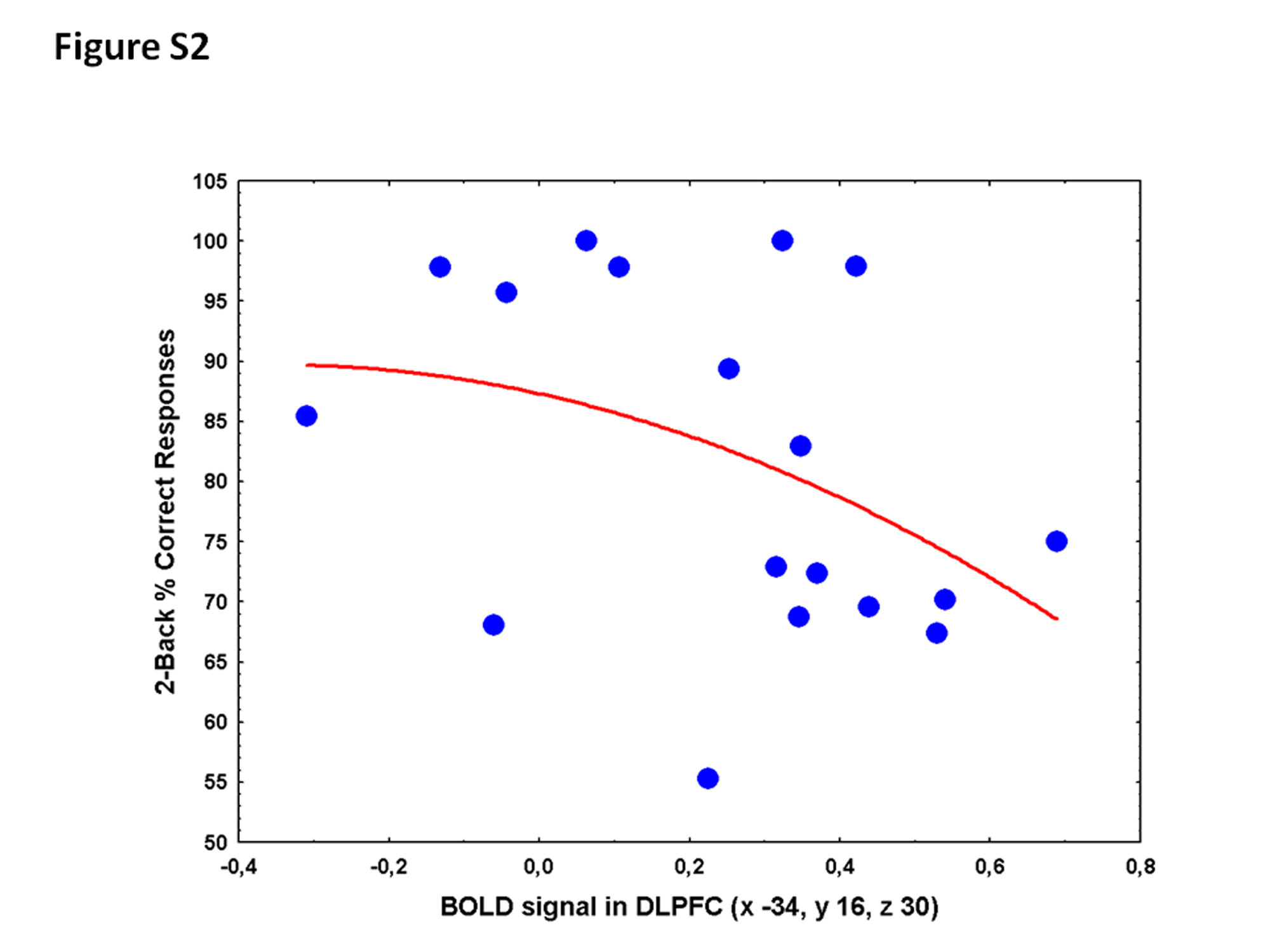

Supplement: Figure S2 — Relationship between behavioral performance and prefrontal activity. Scatterplot of the non-linear relationship in GG subjects between working memory behavioral performance and prefrontal activity during working memory as measured with BOLD fMRI. (0.50 MB TIF) [file pone.0009348.s004.tif]
